# *Streptococcus mutans* copes with heat stress by multiple transcriptional regulons modulating virulence and energy metabolism

**DOI:** 10.1038/srep12929

**Published:** 2015-08-07

**Authors:** Chengcheng Liu, Yulong Niu, Xuedong Zhou, Xin Zheng, Shida Wang, Qiang Guo, Yuqing Li, Mingyun Li, Jiyao Li, Yi Yang, Yi Ding, Richard J. Lamont, Xin Xu

**Affiliations:** 1 State Key Laboratory of Oral Disease, West China Hospital of Stomatology, Sichuan University, Chengdu, PR China; 2 Department of Operative Dentistry and Endodontics, West China Hospital of Stomatology, Sichuan University, Chengdu, PR China; 3 Key Lab of Bio-resources and Eco-environment of Ministry of Education, College of Life Sciences, Sichuan University, Chengdu, PR China; 4 Department of Periodontics, West China Hospital of Stomatology, Sichuan University, Chengdu, PR China; 5 Center for Oral Health and Systemic Disease, School of Dentistry, University of Louisville, Louisville, Kentucky, USA

## Abstract

Dental caries is closely associated with the virulence of *Streptococcus mutans*. The virulence expression of *S. mutans* is linked to its stress adaptation to the changes in the oral environment. In this work we used whole-genome microarrays to profile the dynamic transcriptomic responses of *S. mutans* during physiological heat stress. In addition, we evaluated the phenotypic changes, including, eDNA release, initial biofilm formation, extracellular polysaccharides generation, acid production/acid tolerance, and ATP turnover of *S. mutans* during heat stress. There were distinct patterns observed in the way that *S. mutans* responded to heat stress that included 66 transcription factors for the expression of functional genes being differentially expressed. Especially, response regulators of two component systems (TCSs), the repressors of heat shock proteins and regulators involved in sugar transporting and metabolism co-ordinated to enhance the cell’s survival and energy generation against heat stress in *S. mutans*.

*Streptococcus mutans*, the major etiological agent in dental caries, colonizes the multispecies microbial biofilms that adhere to tooth surfaces^1^,^2^. Sophisticated environmental adaptation is critical for the survival and prevalence of *S. mutans* in the oral cavity, which is a dynamic habitat subject to a wide range of harsh and rapidly changing physiological conditions, including extreme acidity, fluctuation of nutrients, osmotic stress, shifts in temperature and reactive oxygen species, etc^3^.

The adaptation mechanism to heat stress in bacteria is one of the most conserved biological stress responses in bacteria as they adapt to changing environments^4^. Temperature shift signals feed into the transcriptional regulatory systems of bacteria, which affects the physiological changes that enable organisms to adapt and survive^4^. Within the process there are the phases of recognition and adjustment that are coordinated by enzyme and gene level regulation^5^. Alternative sigma factors play an important role in the adaptation process, which is mediated by complex regulatory networks^6^. The *Escherichia coli* heat shock response is highly specific and mediated by members of the sigma 32 regulon through two feedback loops: DnaK/J/GrpE and GroEL/S chaperone systems and inner membrane protease FtsH^7^. Conversely, although the heat stress response of the Gram-positive bacterium *Bacillus subtilis* partially overlaps with that of *E. coli*, it is mainly controlled by the sigma B and CtsR regulators^8^.

Over many years, the effect of environmental stimuli and the responses to them in streptococci, especially *S. mutans*, have attracted major research efforts^3^. *S. mutans* can resist acid stress through a variety of mechanisms including up-regulation of the proton-translocating F-ATPase^3^, elevation of cytoplasmic pH through the agmatine deiminase system (AgDS)^9^ or malolactic fermentation^10^ and reorganization of membrane fatty acid composition^11^. The inactivation of reactive oxygen species (ROS) via enzymes results in resistance to oxidative stress^3^,^12^. Molecular chaperones, the Clp proteolytic system, TCSs, CcpA-dependent and -independent carbon catabolite repression and quorum-sensing (QS) signalling function to facilitate the ecological fitness of *S. mutans*^13^. In addition, protein folding and degradation in *S. mutans* under heat stress are maintained via the involvement of HrcA and CtsR regulons^8^. Due to *S. mutans* lacking an alternative sigma factor^14^, the transcriptional regulatory responses of *S. mutans* to heat stress should be different from *E. coli* and *B. subtilis*. The current study used whole-genome microarrays, in combination with biological analyses, to investigate the transcriptional and metabolic alterations in *S. mutans* during heat stress. The aim was to explore how the metabolic pathway-related genes are regulated by the corresponding transcription factors, and how the metabolism of *S. mutans* changes in response to heat stress.

## Results

### *S. mutans* Shows Dynamic Transcriptomic Responses during Heat Stress

**Figure 1:**
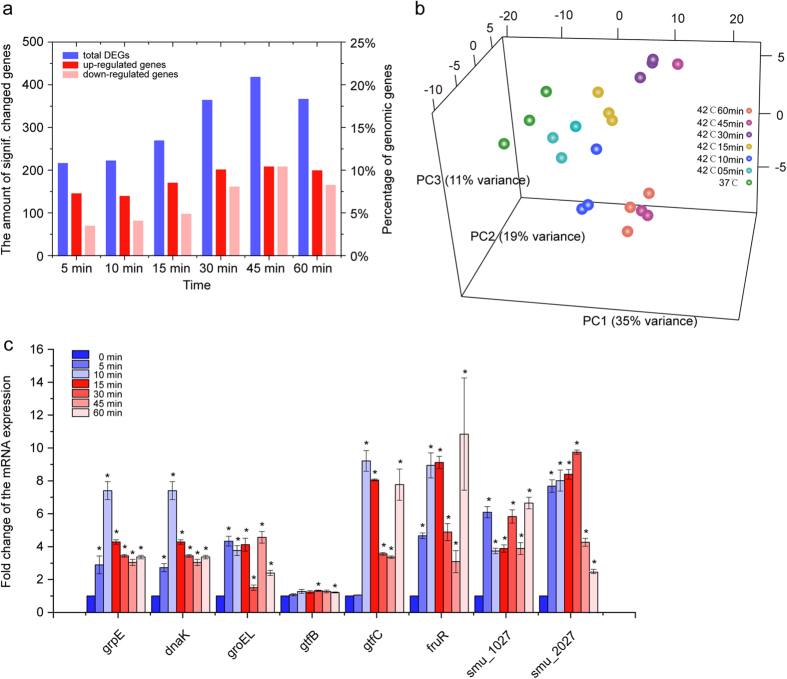
Transcriptional response pattern of *S. mutans* during heat shock. (**a**). Amount of significantly up- and/or down-regulated genes within different heat shock (42 °C) durations. (**b**). Transcriptional patterns of *S. mutans* that had been exposed to heat stress showing clear divergence as determined by principle component analysis (PCA). (**c**). Validation of microarray data by *q*RT-PCR. The expression of eight genes were validated by *q*RT-PCR (n ≥ 3, *P *< *0.05).

To profile the dynamic transcriptomic responses of *S. mutans* during heat stress, we employed whole-genome microarrays for *S. mutans* cells incubated at 37 °C *versus* 42 °C for 5 min, 10 min, 15 min, 30 min, 45 min and 60 min. This resulted in 694 genes that showed differential expression (34.8% of genomic genes) after heat stimuli in *S. mutans*, including many transcriptional regulators, heat shock-related proteins, ATP-binding cassette (ABC) transport family proteins and phosphoenolpyruvate-sugar phosphotransferase (PTS) system family proteins (Table S1). The levels of differentially expressed genes (DEGs) at each time point are shown in Fig. 1a. Between the six time-points, 75 genes in total were expressed differentially (Table S1). Genes involved in the regulation of transcription predominated the 75 “core DEGs”, such as *smu_1027*, *smu_144c* and *rpoE,* which were followed by members of heat shock regulons, including *hrcA*, *groES*, *grpE*, *clpP* and *clpE*. In addition, genes related to DNA metabolism and repair, specifically DNA topoisomerase I (*smu_1002*) and DNA mismatch repair protein (*smu_44*), were induced significantly (Table S1).

Principal component analysis (PCA) was used to determine the distance of transcriptomes corresponding to the six time points. Samples marked with distinct colours were projected to a three-dimensional space (Fig. 1b), and the first principle component (PC1) which had the largest variance (35%) separated the samples the most. The samples spread out into three directions (except for one sample in 42 °C at 45 min), demonstrating most of the differences between the gene expression patterns of *S. mutans* at the six time points. Interestingly, distances from samples in 37 °C increased with the increment of heat stimuli duration. For example, samples grown at 42 °C for 45 min and 60 min were further from ones at 37 °C relative to samples at 42 °C for 5 min and 15 min. Taken together, the PCA plot further demonstrated the dynamic transcriptomic responses of *S. mutans* during heat stress.

To further validate the reliability of the microarray data, the expression of eight genes during heat stress was determined by *q*RT-PCR analysis (Fig. 1c). Most of the genes exhibited consistent patterns of differential expression in both the *q*RT-PCR and microarray, indicating a good concordance of both methods. It should be noted that *dnaK* and *groEL,* which showed near 1.2 fold changes under heat stress based on the microarray data, were up-regulated more than 1.5-fold according to the *q*RT-PCR analysis (Table S1 and Fig. 1c). This could be caused by the underestimation of the fold changes by microarray analysis and the higher sensitivity of *q*RT-PCR^15^. In addition, we further examined the expression of these genes in another *S. mutans* strain (ATCC 55677^TM^) by *q*RT-PCR. Both strains (UA159 and ATCC 55677^TM^) showed similar expression pattern among those selected genes (Fig. S1).

**Figure 2:**
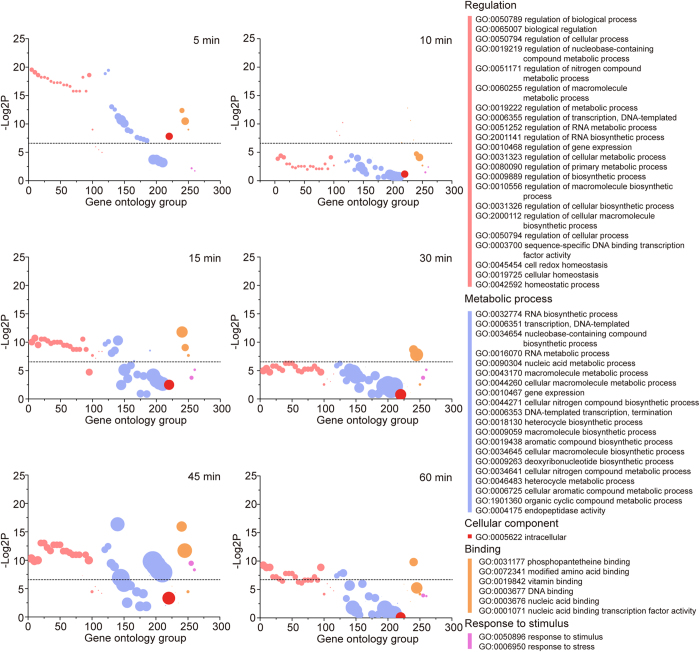
Gene ontology (GO) analysis of differentially expressed genes under heat stress. The significant GO groups were plotted. The dotted line corresponds to a P value of 0.01. The mapping colours indicate GO categories with their names corresponding to the GO group number of the z-coordinate. The number of DEGs included is represented by the circle sizes.

To obtain an overall insight into the impact of heat stress on *S. mutans*, the DEGs from each time point were assigned a function based on a gene ontology (GO) classification. We obtained 52 significantly changed GO terms (P < 0.01) (Table S2). The majority of DEGs with annotated function were related to regulation and metabolic processes. A significant part of these GO terms showed time dependency (Fig. 2), which is related to the amount and distribution of DEGs at each time point.

GO analysis indicated the essential functional gene groups at each time point; however, it only provides the broad gene functional categories. Therefore, to further analyse the induced genes during the early and late heat stress responses in *S. mutans*, subsequent DEGs analysis was performed (Table S3). We noted 22 genes that showed elevated expression only at the 5 min exposure to heat stimuli. These included *ComE*, superoxide dismutase and 12 hypothetical proteins, which suggested their potential role in sensing the environmental signals. In addition, the expression of 33 and 25 genes was significantly induced from 10 and 15 min, respectively. Limitations in the ability of the microarray to detect the gene expression, so genes with volatile expression were not taken into consideration here.

### Multiple Transcription Regulators are Induced in *S. mutans* Exposed to Heat Stimuli

**Figure 3:**
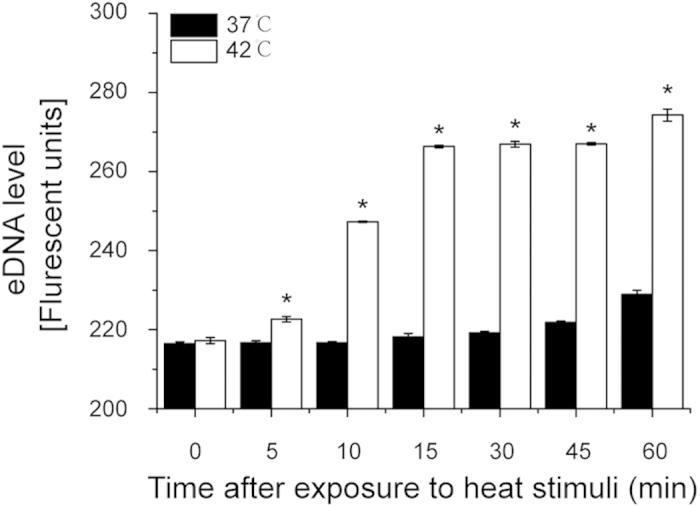
Measurement of eDNA in the planktonic culture of *S. mutans*. eDNA release was significantly induced at 42 °C relative to 37 °C at corresponding time points. *P *< *0.05.

Gene expression in response to changes in the environment is regulated by a wide range of regulatory proteins in bacteria. Among these, two major types of regulators of transcription are the alternative sigma factors and TCSs^6^. A possible alternate sigma factor, i.e. ComX, and more than 100 transcriptional regulators have been identified in the genome of *S. mutans*^14^. A list of 130 transcriptional regulators and their expression in *S. mutans* in response to heat stress are shown in Table S4, including 40 regulators embodied in the RegPrecise database^16^, 15 response regulators of TCSs and 75 other genes annotated as transcriptional regulators in the KEGG database or reported in a previous study^17^. Of these, 66 were observed in this study to have differential expression when exposed to heat stress. These contained 36 transcriptional regulators that showed significantly differential expressions at more than three time points, including alternative sigma factor (*comX*), response regulators of TCSs (*vicR*, *ciaR* and *scnR*) and global regulators (*rpoE* and *fruR*). In parallel with the induction of these global regulators, we also observed an up-regulation of gene encoding autolysin (*smu_704c*) (Table S1) and an increase of eDNA released by *S. mutans* (Fig. 3), indicating an enhanced autolysis during heat stress.

The *smu_2027*, which was reported as a LexA-like regulator being induced under different stresses including heat, oxidation and ofloxacin antibiotic and responsible for regulating tolerance toward DNA damage in a noncanonical SOS mechanism^18^, was also observed significantly up-regulated in the current study (Table S1). In addition, *smu_1027* and *smu_144c* which were annotated as response regulators in the KEGG database also up-regulated upon heat stress (Table S1). Via sequence blast analysis, *smu_144c* were determined to be conserved in most *Streptococci*, while *smu_1027* was unique in *S. mutans*.

### Classic Molecular Chaperones and Proteases Facilitate Heat Fitness of *S. mutans*

**Figure 4:**
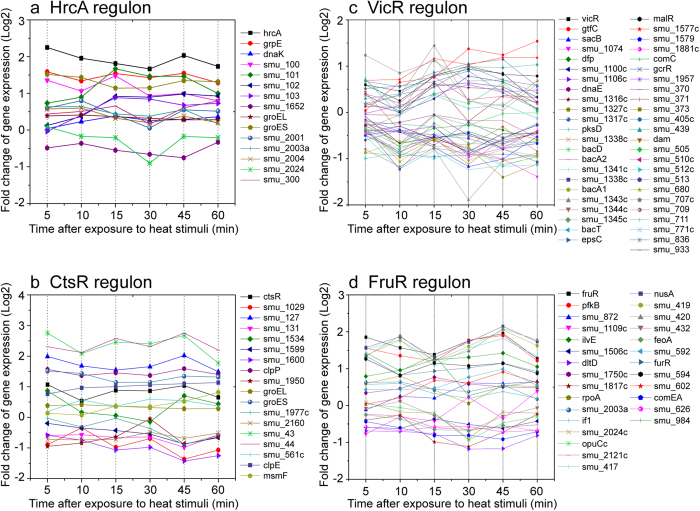
Transcriptional profiles of genes modulated by selected transcription factors under heat stress in *S. mutans*. Line graphs show the normalized expression profiles of DEGs regulated by HrcA (**a**), CtsR (**b**), TCS response regulator VicR (**c**) and global regulator FruR (**d**) at 5, 10, 15, 30, 45, and 60 min at 42 °C relative to 37 °C.

A set of genes that are all regulated by a specific transcription factor are referred to as a regulon, and these are known to control the responses of bacteria to environmental changes^19^. To further decipher how the metabolic pathway genes are regulated by the corresponding transcription factors, we analysed the target genes of these differentially expressed transcriptional regulators. From this, the regulon information of nine differentially expressed transcriptional regulators was obtained (Table S5). Of the regulons, two (HrcA and CtsR) have been previously determined to have critical functions in modulating protein folding and degradation pathways in Gram-positive bacteria under environmental stress^20^,^21^. They control the expression of members of two well-defined families of heat shock proteins, i.e. molecular chaperones and proteases^21^,^22^. Our microarray data showed that the HrcA and CtsR regulons were induced in *S. mutans* under heat stress (Fig. 4a, b). Up-regulated operons controlled by HrcA under heat stress included *grpE-dnaK*, *groEL-groES* and *smu_100–103* (Fig. 4a). Although microarray analysis showed no significant change in expression of *dnaK* and *groEL* during heat stress, the expression of these genes were demonstrated to be up-regulated by *q*RT-PCR analysis. The expressions of both *dnaK* and *groEL* were also demonstrated to be up-regulated after heat stress in *S. mutans* by slot blot analysis in previous studies^20^,^22^. Unlike most Gram-positive bacteria, in *S. mutans* the HrcA and CtsR repressors dually control the transcription of the *groE* operon^23^. In addition, other genes regulated by CtsR are up-regulated after exposure to 42°C, including gene encoding ATP-dependent protease (*clpE*), ATP-dependent Clp protease proteolytic subunit (*clpP*)^21^,^24^ and genes related to DNA mismatch repair (*smu_43* and *smu_44*) (Fig. 4b). ClpP is proteolytic subunit of Clp functional complex, which specifically targets damaged and misfolded proteins. ClpP alone can also degrade proteins that might be toxic for the bacteria but with relatively lower efficiency^21^,^24^. Recent study showed that deletion of *clpP* in *S. mutans* resulted in a sensitive phenotype to heat stress^24^. Hence, the up-regulation of ClpE and ClpP is believed to be a defensive mechanism adopted by the bacterium to clear damaged or misfolded proteins under heat stress. Heat shock proteins and proteases were also reported to be related to acid tolerance in *S. mutans*^20^,^24^. We thus examined whether pre-adaptation to heat stress would affect bacterial survival under acid challenge. We observed that pre-adaptation to heat stress had no significant effect on cell survival under acid challenge (Fig. S2), as also being reported by others^25^.

### Essential Response Regulators of TCSs in *S. mutans* upon Heat Stress

Bacterial TCS transduction systems play important roles for many bacteria by enabling them to detect and respond to diverse changes/stresses in the environment. TCS response regulators have been shown to be involved in regulating target genes in *S. mutans* under acid stresses^26^.

VicR is the response regulator of the vicKR TCS system, which is essential for the survival of *S. mutans* and plays a major role in the stress response, competence development, sugar metabolism and biofilm formation^27^. A previous study found that *vicR* was an essential response regulator of *S. mutans* upon treatment with carolacton (a biofilm inhibitor) by regulating the expression of *gtfB*, *gtfD* and *gbpB*^28^. In this work, we observed a set of genes, including gene encoding glucosyltransferase-SI (*gtfC*), transcriptional regulators (*malR* and *smu_439*), gene encoding endolysin (*smu_707c*) and UDP-N-acetylglucosamine 2-epimerase (*epsC*), were regulated by *vicR* in *S. mutans* under heat stress (Fig. 4c).

Of the TCSs, CiaR is a global regulator involved in multiple stress responses, biofilm formation and bacteriocin production in *S. mutans*^19^. Genes such as *smu_139*, *smu_239* and *smu_739* have previously been reported as potential targets of CiaR^19^. Under heat stress, a significant continuous up-regulation of *smu_139* was observed, along with increased expression of *ciaR* at multiple time points (5 min, 15 min and 45 min) after exposure to heat stimuli (Table S5). The *smu_139* gene and its role in *S. mutans* stress response should be further investigated as it has been predicted to encode a protein associated with carbohydrate metabolism in the KEGG database^29^.

### Heat Stimuli Induce Specific Sugar Transport Regulons and Glucans Synthesis

The regulons of the sugar-specific transcriptional regulators were significantly induced upon heat stress in *S. mutan*s, such as the repressors FruR, GalR, MalR and LacR (Fig. 4d and Table S5). Although DEGs were enriched in these predicted regulons, only two genes encoding the EII loci (fructose-1-phosphatate kinase and galactose-6-phosphate isomerase subunit LacA) were induced significantly, indicating that heat may induce other biological processes controlled by these sugar-specific transcriptional regulators. This confirms previous studies in which PTSs were shown to be related to transcriptional regulation, catabolite repression and enzyme activity as well as sugar transport and phosphorylation^30^. Instead, heat activated the expression of *smu_100–103*, which were predicted to be the target genes of HrcA. A previous study has shown that this operon was a sucrose-inducible PTS and it might transfer carbohydrates synthesized by glucosyltransferase-I (GtfB) and glucosyltransferase-SI (GtfC) of *S. mutans*^31^. Their transcriptional regulator, *smu_105*, was not affected by heat stress. Collectively, the results indicated that this sucrose-inducible PTS could also be induced and might be involved in glycogen uptake under heat stress. In *S. mutans,* sugar substrates can also be taken up by ABC transporters. Under heat stress, we observed the up-regulation of two members of sugar ABC transporter systems, i.e. polysaccharide ABC transporter permease (*smu_827*) and the multiple sugar-binding ABC transporter permease MsmF (*smu_879*) (Table S1).

**Figure 5:**
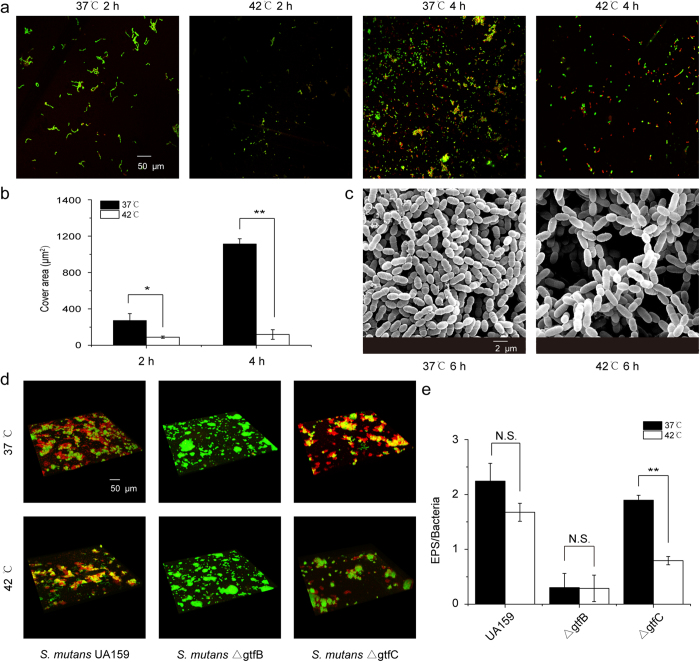
Sucrose-dependent initial attachment and biofilm architecture of *S. mutans* during heat shock. (**a**). Representative images of *S. mutans* (live: green; dead: red) grown on the glass surface after 2 h and 4 h incubation at 37 °C and 42 °C, respectively. (**b**). Quantitative analyses of coverage area (μm^2^) of *S. mutans* grown on the glass surface. (**c**). Representative SEM images from 6 h *S. mutans* biofilms at 37 °C versus 42 °C. (**d**). Representative confocal laser scanning microscopy images of EPS (red) produced by *S. mutans* UA159 and its *gtf-*mutants (green) under heat stress. (**e**). Quantitative analyses of normalized EPS (EPS/bacteria) produced by *S. mutans* UA159 and its *gtf-*mutants. Data were obtained from an average of three independent experiments and shown as mean ± standard deviation. *P < 0.05, **P < 0.01 compared with 37 °C controls at corresponding time points. N.S.: no significant difference.

Sugars are microbial substrates for the synthesis of glucan, which is required for adherence and biofilm formation in *S. mutans*^32^. Under heat stress, *gtfC* and *gbpC*, but not *gtfB*, *gtfD*, *gbpB*, *gbpD* and *ftf*, were identified to be up-regulated by more than 1.5 fold from 10 min to 60 min (Table S1). Further investigation of the adherence and biofilm formation of *S. mutans* by fluorescent live/dead staining showed that the initial attachment was significantly impaired during heat stress (Fig. 5a,b). SEM analysis further confirmed that biofilm formation was compromised, as the 6 h biofilms showed more channel-like structures at 42 °C relative to those at 37 °C (Fig. 5c). As *S. mutans* GtfC can produce both water-insoluble and water-soluble glucans^33^, the up-regulation of *gtfC* may compensate the compromised sugar-dependent biofilm formation under heat shock. We thus quantified the extracellular polysaccharides (EPS) synthesized by *S. mutans* UA159, *gtfB-* and *gtfC*-deficient mutants with fluorescence-labelled dextrans. We found that EPS generation by *S. mutans* UA159 strain was not significantly impaired at 42 °C relative to 37 °C (Fig. 5d,e), indicating possible compensatory mechanisms of this bacterium under heat stress. The *gftC*-deficient mutant, but not the *gtfB*-deficient mutant, showed significantly impaired EPS production at 42 °C relative to 37 °C (Fig. 5d,e), further supporting our speculation that up-regulation of *gtfC* may involve the compensatory EPS-generation by *S. mutans* after heat shock.

### *S. mutans* Up-regulates ATP Turnover to Counter Heat Stress

**Figure 6:**
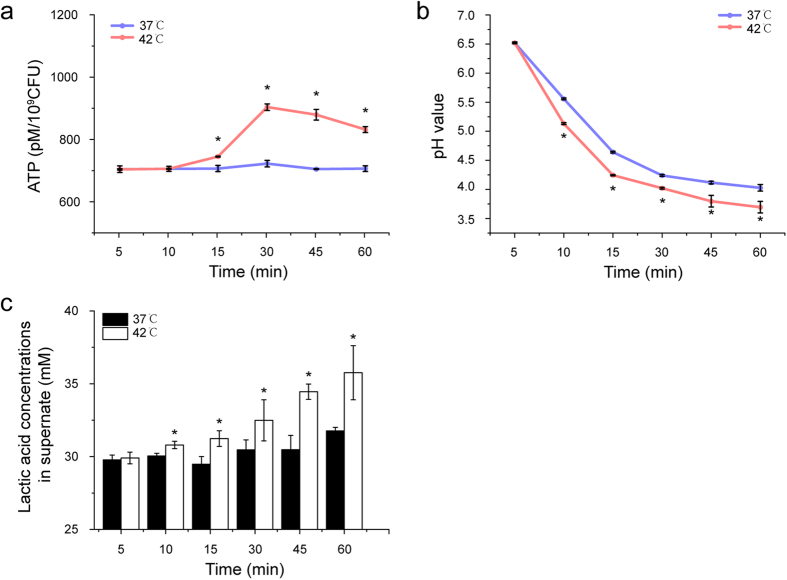
Effect of heat stress on metabolism in *S. mutans*. (**a**). Time-course of intracellular ATP level of *S. mutans* incubated at 37 °C versus 42 °C. (**b**). Glycolysis-induced pH drop by *S. mutans* grown in sucrose-rich medium incubated at 37 °C versus 42 °C. (**c**). Quantification of lactic acid generated by *S. mutans* incubated at 37 °C versus 42 °C. Results are presented as mean ± standard deviations (n = 3). *P < 0.05 level compared with 37 °C controls at corresponding time points.

To survive under heat stress, *S. mutans* up-regulated genes encoding ATP-dependent transporters together with heat-triggered proteins and genes related to DNA-repair machinery (Table S1). All these biological processes require increased ATP turnover during the heat shock process. Here we observed an increase of intracellular ATP levels in *S. mutans* during heat stress (Fig. 6a). In parallel with increased intracellular ATP, glycolysis by *S. mutans*, as reflected by a pH drop and the amount of lactic acid produced in the sucrose-rich medium, was also significantly enhanced during heat shock (Fig. 6b,c). In addition, genes encoding fructose-1-phosphate kinase (*smu_871*) and NADH oxidase (*nox*), respectively, were continuously up-regulated under heat stress (Table S1). Fructose-1-phosphate kinase, also known as phosphofructokinase, is one of the three regulated enzymes in glycolysis^34^, and NADH oxidase is an enzyme critical for the maintenance of the NADH^+^:NAD^+^ ratios during active glycolysis^35^. That glycolysis is increased under heat stress in *S. mutans* is further shown by these two genes being up-regulated.

## Discussion

The ability to respond to environmental perturbation is critical for bacteria to colonise new areas and survive^36^. The strategy employed by bacteria to tolerate heat stress is highly conserved^36^, and the accumulation of unfolded polypeptides as well as impairment of DNA act as triggers that lead to various changes in the global transcriptional profiles at different time points^4^. The specific mechanisms in the heat stress response differ between Gram-positive and Gram-negative bacteria^8^,^37^. Unlike the classic Gram-positive model bacterium *B. subtilis*, *S. mutans* is characterized by a host-associated lifestyle, non-sporulating phenotype and lower GC content (less than 40%). This has led to the recent proposal to consider *S. mutans* as a new Gram-positive paradigm^2^. Considering its ‘feast or famine’ lifestyle, strong aciduricity and relatively small genome, *S. mutans* has most likely developed unique measures to cope with heat stress. Our data showed that *S. mutans* had a dynamic transcriptional response to heat shock.

*S. mutans* has an even and steady response to heat shock as indicated by a balanced distribution of DEGs at multiple time points. This pattern of response is distinct from that of *E. coli*, whose DEGs peaked within 10 min upon heat shock^38^. The mild heat shock response (42 °C) of *E. coli* is almost entirely regulated by the amount and activity of the alternative sigma factor (Sigma 32, RpoH)^39^. Conversely, *B. subtilis* responds to general stress including heat stress via sigma B^40^. In addition, *B. subtilis* also possesses multiple sigma factors to cope with various environmental stimuli^41^. Unlike *E. coli* and *B. subtilis*, *S. mutans* UA159 possesses only one copy of a possible alternate sigma factor, i.e. ComX^14^. However, more than 100 transcriptional regulators (whole genome of 2 Mb with 1900 genes) and 14 TCSs, as well as one orphan response regulator have been identified in the genome of this bacterium^14^,^42^. In this work, 66 transcriptional regulators (including comX) were induced under heat stimuli in *S. mutans*. Moreover, the regulons analysis showed significantly induced target genes of multiple transcriptional regulators upon heat shock. Our data indicated that many transcriptional regulators coordinate the fitness of *S. mutans* to heat stress, possibly due to the lack of alternative sigma factors in the bacteria. In addition, the up-regulated expression of *comX* and *comYABCD* at multiple time points may increase the natural competence of *S. mutans* under heat stress. The heat-induced up-regulation of *comX* and *comYABCD* may justify the rationale of a heat shock method for bacterial transformation^43^.

Heat shock proteins (HSPs), which are similar in both eukaryotic and prokaryotic cells, are vital for cells to survive heat stress^44^,^45^. Members of two well-defined families of heat shock proteins, i.e. molecular chaperones and proteases, were induced during heat shock in *S. mutans.* Specifically, the genes encoding ClpE and ClpP were significantly induced in the current study. The ClpP protease is associated with ClpE to form a functional complex that is critical for the survival of *S. mutans* under environmental stress, including acid, temperature, and oxidative stresses by degrading denatured proteins or stabilizing native proteins^21^,^24^,^46^. *S. mutans* that was deficient in *clpP* was more sensitive than the wild-type to environmental stimuli^47^. Hence, the observed up-regulation of ClpE and ClpP during heat stress may contribute to the fitness of *S. mutans* under this harsh condition.

Notably, the proper function of these heat shock proteins will inevitably consume extra energy during heat stress. To cope with the energy demand, *S. mutans* may increase production of ATP. Here we observed an elevated level of intracellular ATP, similar to that reported in *Staphylococcus aureus* during heat shock^48^. Elevation of intracellular ATP may be the consequence of either enhanced bacterial energy-generation or energy-sparing adaptations (e.g. growth arrest). The observed enhanced microbial glycolysis, lactic acid production and up-regulation of genes encoding NADH oxidase and sugar transporters in the current study suggest that *S. mutans* may more likely up-regulate energy generation to cope with heat shock.

Heat stress also attenuated the initial attachment of *S. mutans* and led to the formation of a biofilm that contained more channels. The sucrose-dependent attachment of *S. mutans* was mediated by two major types of adhesions, i.e. cell-surface proteins and sucrose-derived glucans^49^. GtfB predominately generates water-insoluble polysaccharides, which act as the major components of the extracellular matrix (ECM) and mediate cellular adherence and biofilm formation of *S. mutans*^50^. On the contrary, GtfC is related to the synthesis of a glucan that has a low-molecular-mass and is partially water-soluble, and this can be either used to produce energy via being metabolized or to form part of a biofilm^50^. Here we observed that *gtfC* but not *gtfB* was up-regulated during heat shock. The up-regulation of *gtfC* may metabolize carbohydrate substrates for both energy-generation and EPS generation. Our data that *gtfC-*mutant instead of *gtfB*-mutant demonstrated significantly impaired EPS generation at 42°C relative to 37 °C further support the compensatory role of *gtfC* in EPS generation during heat stress. The up-regulation of *gtfC* may further help to offset damaging stresses via molecular machinery (e.g. HSPs) that consumes ATP. In addition, GtfC can be directly embedded in the acquired pellicle with enzymatic activity^51^. The partially soluble glucans can be recognized by other subtypes in the cell surface, such as glycosyltransferases (Gtfs) or glucan binding proteins (Gbps), thus contributing to cell attachment^51^. In addition to *gtfC, gbpC* was also up-regulated by heat stress. This may also compensate for the compromised biofilm formation under heat shock by forming a less energy consuming structure of GtfC-glucan-GbpC.

Taken together, by using whole-genome microarrays to profile the transcriptomic responses of *S. mutans* during heat shock, we have demonstrated that *S. mutans* acts as a new paradigm of a Gram-positive bacterium that responds to heat stress in a distinct pattern. The dynamic transcriptional alterations of multiple regulators and functional genes, together with enhanced glycolytic activity, attenuated sucrose-dependent initial attachment and impaired biofilm architecture, indicate metabolic adaptations by this bacterium to compensate for the extra energy demand required to counter adverse environmental stimuli.

## Materials and Methods

### Bacterial Strains and Growth Conditions

The *S. mutans* UA159 and *S. mutans* ATCC 55677^TM^ strains were obtained from the American type Culture Collection (ATCC, Manassas, VA, USA). The *gtfB*- and *gtfC*-deficient mutants were kindly provided by Robert A. Burne (Department of Oral Biology, College of Dentistry, University of Florida, Gainesville, FL). *S. mutans* was routinely grown in brain heart infusion broth (BHI; Difco, Sparks, MD, USA) at 37 °C anaerobically^52^. To enable the sucrose-dependent initial attachment and biofilm assay, the BHI broth was supplemented with 1% sucrose. For the exposure to heat stress, cultures of *S. mutans* (OD_600 nm _= 0.5) were transferred from 37 °C to a 45 °C water bath, and the temperature of each culture was raised to 42 °C in <4 min. Then the cultures were transferred to a 42 °C water bath for 5 min, 10 min, 15 min, 30 min, 45 min and 60 min. The control was a cell culture grown at 37 °C and collected before the transfer to the 45 °C water bath.

### Microarray Procedures

Immediately after heat stress, all cell cultures were collected and treated with RNA protect reagent (Qiagen, Valencia, CA, USA). A previously described RNA extraction method was used^52^. In brief, RNA was extracted and purified using RNeasy Mini kits (Qiagen) and digested with RNase-free DNase I (Qiagen). The concentration of RNA was measured by a Nanodrop ND 1000 spectrophotometer (Thermo Fisher Scientific, Pittsburgh, PA, USA). Agarose electrophoresis was then used to determine the integrity and quality of RNA.

*S. mutans* UA159 whole-genome arrays (8 × 15 K) were obtained from Agilent and included 1997 probes for *S. mutans* transcripts. RNA samples were labelled with cyanine 3 or cyanine 5 (GE Healthcare), and the labelled RNA was hybridized to the microarray at 45 °C overnight. The arrays were scanned with an Agilent G2562CA Microarray Scanner, and an Agilent Feature Extraction Software (Version 11.0.1.1) was used to analyse the acquired images. A range of R/Bioconductor packages were used with the raw data to normalize and annotate it. DEGs were identified by the Rank Product method, with fold changes >1.5 and P < 0.05^53^. Gene set enrichment analysis (GSEA) was employed to determine the significant GO terms of the DEGs^54^. The microarray data were confirmed using *q*RT-PCR with the expression of eight transcripts using a previously published method and the primers in Table S6^52^,^55^. By integrating the transcript units information^56^, promoter binding motifs^28^, and experimental validation, we summarized heat stress-induced regulons of *S. mutans* (see Table S5 for details).

### Microarray Data Accession

All data obtained from the microarray analysis have been added to the NCBI Gene Expression Omnibus database with an accession number GSE59302 (http://www.ncbi.nlm.nih.gov/geo/query/acc.cgi?token=abetgqeadtavhcv&acc=GSE59302).

### ATP Determination, Glycolytic pH Drop Assay, Lactic Acid and eDNA determination

To measure the influence of heat stress (42 °C) on intracellular ATP levels, mid-logarithmic phase (OD_600 nm _= 0.5) cell cultures of *S. mutans* were collected after exposure to heat stimuli at 0 min, 5 min, 10 min, 15 min, 30 min, 45 min and 60 min. ATP levels were quantified by a luminometric ATP detection assay with BacTiter-GloTM kit from Promega as described in a previous study^48^. To determine the effect of heat stress on the glycolysis of *S. mutans*, the decrease in the pH over 75 min in a sucrose solution (1%, wt/vol) was recorded^57^. For lactic acid determination, supernatants of *S. mutans* cultures were collected by centrifugation (4000 rpm, 10 min) after incubation at 37 °C or 42 °C for 5 min, 10 min, 15 min, 30 min, 45 min and 60 min, respectively. Lactate concentrations in the supernatants were determined using an enzymatic (lactate dehydrogenase) method previously described^58^. The absorbance at 340 nm (OD_340 nm_) before and after the reaction was measured by a microplate reader (Gene, Hong Kong, China). Standard curves were plotted using a lactic acid standard (Supelco Analytical, Bellefonte, PA).

eDNA in planktonic cultures was measured by a spectrofluorometry method as previously reported with minor modifications^59^. Briefly, cell cultures of *S. mutans* UA159 with an OD_600 nm_ value of 0.5 were collected and incubated in a water bath (37 °C or 42 °C). At the indicated time points, 4.5 ml of supernatant were collected by centrifugation (12,000 rpm, 1 min) and filtered by DNA purification columns (Qiagen). eDNA was then eluted by 210 μl H_2_O and labelled with 2.5 μl of 50 μM cell-impermeant fluorescent dye SYTOX Green (Invitrogen) for 10 min. 200 μl of stained samples were transferred to a 96-well plate (Corning, Inc., NY, USA) and eDNA was quantified by measuring the intensity of fluorescence (excitation at 485 nm, and emission at 535 nm) using a BioTek Synergy 2 HT microplate reader (Biotek Instruments, Winooski, VT, USA).

### Sucrose-dependent Initial Attachment and Biofilm Assay

The effect of heat stress on the attachment of *S. mutans’* cells was determined using a previously described method^60^. *S. mutans* was collected at mid-log phase, washed twice with PBS, and then re-suspended in BHI medium containing 1% sucrose to a level of 1 × 10^6 ^CFU/ml. After 2 h or 4 h incubation on saliva pre-coated coverslips, biofilms were collected and stained with the fluorescent Live/Dead Backlight^TM^ stain (Molecular Probes Inc., Eugene, Oregon, USA), and observed with a Leica TCS SP2 confocal laser scanning microscope (Leica, Germany). Images were analysed by Image-pro Plus 6.0 (Media Cybernetics Inc., Bethesda, MD, USA) to quantify the amount of initially attached bacteria. The heat stress effect on the biofilm structure was further determined using a scanning electron microscope (SEM) as described previously^52^. To further investigate the EPS synthesis of *S. mutans* under heat stress, 12 h biofilm of *S. mutans* UA159, and its *gtfB-*/*gtfC*-deficient mutants grown on saliva pre-coated coverslips were double-labelled with 2.5 μmol L^−1^ of Alexa Fluor 647-labelled dextran conjugate (10000 MW; absorbance/fluorescence emission maxima of 650/668 nm; Molecular Probes Inc., Eugene, OR, USA) and a SYTO 9 green fluorescent nucleic acid stain (2.5 μmol L^−1^, 480/500 nm; Molecular Probes Inc.) as described previously^52^.

### Cross-protection assay

Cross-protection experiments were performed to determine whether pre-adaption to heat stress (42 °C, 2 h) could affect the survival of *S. mutans* challenged at acidic condition (pH = 2.8, 2 h). Briefly, *S. mutans* cells at mid-exponential phase (OD_600nm _= 0.5) were incubated in BHI medium (pH = 7) at either 37 °C or 42 °C for 2 h, and then re-suspended in 0.1 M sodium citrate buffer (pH = 2.8, 2 h). 200 μl of cell suspend before and after acid challenge were diluted and plated on BHI agar plates (pH 7.5). The viable cells were counted after 48 h incubation (37 °C) and the survival rate after acid challenge was calculated.

### Statistical Methods

Microarray data were analysed using the R Project for Statistical Computing. A two-tailed paired Student’s *t t*est was used for *q*RT-PCR, metabolite determination and biomass assay. Data in the graphs are expressed as the mean ± standard deviation and P values < 0.05 were considered significantly different.

## Additional Information

**How to cite this article**: Liu, C. *et al.* *Streptococcus mutans* copes with heat stress by multiple transcriptional regulons modulating virulence and energy metabolism. *Sci. Rep.* **5**, 12929; doi: 10.1038/srep12929 (2015).

## Supplementary Material

Supplementary Figures

Supplementary Dataset 1

## References

[b1] Decker E. M., Klein C., Schwindt D. & von Ohle C. Metabolic activity of Streptococcus mutans biofilms and gene expression during exposure to xylitol and sucrose. Int J Oral Sci. 6(4):195–204 (2014). 25059251 10.1038/ijos.2014.38 PMC5153587

[b2] Lemos J. A., Quivey R. G. Jr., Koo H. & Abranches J. Streptococcus mutans: a new Gram-positive paradigm? Microbiology. 159, 436–445 (2013). 23393147 10.1099/mic.0.066134-0 PMC4083656

[b3] Lemos J. A., Abranches J. & Burne R. A. Responses of cariogenic streptococci to environmental stresses. Curr Issues Mol Biol. 7, 95–107 (2005). 15580782

[b4] Narberhaus F. Translational control of bacterial heat shock and virulence genes by temperature-sensing mRNAs. RNA Biol. 7, 84–89 (2010). 20009504 10.4161/rna.7.1.10501

[b5] Kotte O., Zaugg J. B. & Heinemann M. Bacterial adaptation through distributed sensing of metabolic fluxes. Mol Syst Biol. 6, 355 (2010). 20212527 10.1038/msb.2010.10 PMC2858440

[b6] Herrou J., Rotskoff G., Luo Y., Roux B. & Crosson S. Structural basis of a protein partner switch that regulates the general stress response of alpha-proteobacteria. Proc Natl Acad Sci U S A. 109, E1415–1423 (2012). 22550172 10.1073/pnas.1116887109 PMC3361416

[b7] Lim B. *et al.* Heat shock transcription factor sigma(32) co-opts the signal recognition particle to regulate protein homeostasis in *E. coli*. PLoS Biol. 11, e1001735, (2013). 24358019 10.1371/journal.pbio.1001735 PMC3866087

[b8] Elsholz A. K., Michalik S., Zuhlke D., Hecker M. & Gerth U. CtsR, the Gram-positive master regulator of protein quality control, feels the heat. EMBO J. 29, 3621–3629 (2010). 20852588 10.1038/emboj.2010.228 PMC2982754

[b9] Griswold A. R., Jameson-Lee M. & Burne R. A. Regulation and physiologic significance of the agmatine deiminase system of *Streptococcus mutans* UA159. J Bacteriol. 188, 834–841 (2006). 16428386 10.1128/JB.188.3.834-841.2006 PMC1347362

[b10] Sheng J. & Marquis R. E. Enhanced acid resistance of oral streptococci at lethal pH values associated with acid-tolerant catabolism and with ATP synthase activity. FEMS Microbiol Lett. 262, 93–98 (2006). 16907744 10.1111/j.1574-6968.2006.00374.x

[b11] Fozo E. M. & Quivey R. G. Jr. Shifts in the membrane fatty acid profile of Streptococcus mutans enhance survival in acidic environments. Appl Environ Microbiol. 70, 929–936 (2004). 14766573 10.1128/AEM.70.2.929-936.2004 PMC348902

[b12] Xu P. *et al.* Genome of the opportunistic pathogen Streptococcus sanguinis. J Bacteriol. 189, 3166–3175 (2007). 17277061 10.1128/JB.01808-06 PMC1855836

[b13] Smith E. G. & Spatafora G. A. Gene regulation in S. mutans: complex control in a complex environment. J Dent Res. 91, 133–141 (2012). 21743034 10.1177/0022034511415415

[b14] Ajdic D. *et al.* Genome sequence of Streptococcus mutans UA159, a cariogenic dental pathogen. Proc Natl Acad Sci U S A. 99, 14434–14439 (2002). 12397186 10.1073/pnas.172501299 PMC137901

[b15] Liang W. D. *et al.* Gene expression profiling of Clostridium botulinum under heat shock stress. Biomed Res Int. 2013, 760904 (2013). 24195079 10.1155/2013/760904 PMC3806222

[b16] Novichkov P. S. *et al.* RegPrecise 3.0–a resource for genome-scale exploration of transcriptional regulation in bacteria. BMC genomics. 14, 745 (2013). 24175918 10.1186/1471-2164-14-745 PMC3840689

[b17] Xue X. L., Tomasch J., Sztajer H. & Wagner-Dobler I. The Delta Subunit of RNA Polymerase, RpoE, Is a Global Modulator of Streptococcus mutans Environmental Adaptation. J Bacteriol. 192, 5081–5092 (2010). 20675470 10.1128/JB.00653-10 PMC2944538

[b18] Leung V., Ajdic D., Koyanagi S. & Levesque C. M. The formation of Streptococcus mutans persisters induced by the quorum-sensing peptide pheromone is affected by the LexA regulator. J Bacteriol. 197, 1083–1094 (2015). 25583974 10.1128/JB.02496-14 PMC4336345

[b19] Wu C. *et al.* Regulation of ciaXRH operon expression and identification of the CiaR regulon in Streptococcus mutans. J Bacteriol. 192, 4669–4679 (2010). 20639331 10.1128/JB.00556-10 PMC2937423

[b20] Jayaraman G. C., Penders J. E. & Burne R. A. Transcriptional analysis of the Streptococcus mutans hrcA, grpE and dnaK genes and regulation of expression in response to heat shock and environmental acidification. Mol Microbiol. 25, 329–341(1997). 9282745 10.1046/j.1365-2958.1997.4671835.x

[b21] Lemos J. A. & Burne R. A. Regulation and Physiological Significance of ClpC and ClpP in Streptococcus mutans. J Bacteriol. 184, 6357–6366 (2002). 12399506 10.1128/JB.184.22.6357-6366.2002 PMC151938

[b22] Lemos J. A., Chen Y. Y. & Burne R. A. Genetic and physiologic analysis of the groE operon and role of the HrcA repressor in stress gene regulation and acid tolerance in Streptococcus mutans. J Bacteriol. 183, 6074–6084 (2001). 11567008 10.1128/JB.183.20.6074-6084.2001 PMC99687

[b23] Lemos J. A., Luzardo Y. & Burne R. A. Physiologic effects of forced down-regulation of dnaK and groEL expression in Streptococcus mutans. J Bacteriol. 189, 1582–1588 (2007). 17172345 10.1128/JB.01655-06 PMC1855735

[b24] Hou X. H., Zhang J. Q., Song X. Y., Ma X. B. & Zhang S. Y. Contribution of ClpP to stress tolerance and virulence properties of Streptococcus mutans. J Basic Microbiol. 54, 1222–1232 (2014). 24979467 10.1002/jobm.201300747

[b25] Svensater G., Sjogreen B. & Hamilton I. R. Multiple stress responses in Streptococcus mutans and the induction of general and stress-specific proteins. Microbiology. 146, 107–117 (2000). 10658657 10.1099/00221287-146-1-107

[b26] Tremblay Y. D., Lo H., Li Y. H., Halperin S. A. & Lee S. F. Expression of the Streptococcus mutans essential two-component regulatory system VicRK is pH and growth-phase dependent and controlled by the LiaFSR three-component regulatory system. Microbiology. 155, 2856–2865 (2009). 19589829 10.1099/mic.0.028456-0

[b27] Senadheera M. D. *et al.* A VicRK signal transduction system in Streptococcus mutans affects gtfBCD, gbpB, and ftf expression, biofilm formation, and genetic competence development. J Bacteriol. 187, 4064–4076 (2005). 15937169 10.1128/JB.187.12.4064-4076.2005 PMC1151735

[b28] Sudhakar P. *et al.* Construction and verification of the transcriptional regulatory response network of Streptococcus mutans upon treatment with the biofilm inhibitor carolacton. BMC genomics. 15, 739 (2014). 10.1186/1471-2164-15-362 PMC4048456 24884510

[b29] Kanehisa M. *et al.* From genomics to chemical genomics: new developments in KEGG. Nucleic Acids Res. 34, D354–357 (2006). Available at: http://www.genome.jp/kegg/. (Accessed: 74.0, April 2015). 16381885 10.1093/nar/gkj102 PMC1347464

[b30] Vadeboncoeur C. & Pelletier M. The phosphoenolpyruvate:sugar phosphotransferase system of oral streptococci and its role in the control of sugar metabolism. FEMS Microbiol Rev. 19, 187–207 (1997). 9050218 10.1111/j.1574-6976.1997.tb00297.x

[b31] Ajdic D. & Chen Z. A novel phosphotransferase system of Streptococcus mutans is responsible for transport of carbohydrates with-1,3 linkage. Mol Oral Microbiol. 28, 114–128 (2013). 23193985 10.1111/omi.12009 PMC3593818

[b32] dos Santos N. Relationship among dental plaque composition, daily sugar exposure and caries in the primary dentition. Caries Res. 37, 236–236 (2003). 10.1159/000065959 12399695

[b33] Bowen W. H. & Koo H. Biology of Streptococcus mutans-derived glucosyltransferases: role in extracellular matrix formation of cariogenic biofilms. Caries Res. 45, 69–86 (2011). 21346355 10.1159/000324598 PMC3068567

[b34] Loo C. Y., Mitrakul K., Voss I. B., Hughes C. V. & Ganeshkumar N. Involvement of an inducible fructose phosphotransferase operon in Streptococcus gordonii biofilm formation. J Bacteriol. 185(21), 6241–54 (2003). 14563858 10.1128/JB.185.21.6241-6254.2003 PMC219402

[b35] Baker J. L. *et al.* Streptococcus mutans NADH Oxidase Lies at the Intersection of Overlapping Regulons Controlled by Oxygen and NAD(+) Levels. J Bacteriol. 196, 2166–2177 (2014). 24682329 10.1128/JB.01542-14 PMC4054193

[b36] Mitchell A. *et al.* Adaptive prediction of environmental changes by microorganisms. Nature. 460, 220–224 (2009). 19536156 10.1038/nature08112

[b37] Shenhar Y., Rasouly A., Biran D. & Ron E. Z. Adaptation of Escherichi coli to elevated temperatures involves a change in stability of heat shock gene transcripts. Environ Microbiol. 11, 2989–2997 (2009). 19624711 10.1111/j.1462-2920.2009.01993.x

[b38] Jozefczuk S. *et al.* Metabolomic and transcriptomic stress response of Escherichia coli. Mol Syst Biol. 6, 364 (2010). 20461071 10.1038/msb.2010.18 PMC2890322

[b39] Chakraborty A., Mukherjee S., Chattopadhyay R., Roy S. & Chakrabarti S. Conformational adaptation in the E. coli sigma 32 protein in response to heat shock. J Phys Chem B. 118, 4793–4802 (2014). 24766146 10.1021/jp501272n

[b40] Reder A., Pother D. C., Gerth U. & Hecker M. The modulator of the general stress response, MgsR, of Bacillus subtilis is subject to multiple and complex control mechanisms. Environ Microbiol. 14, 2838–2850 (2012). 22812682 10.1111/j.1462-2920.2012.02829.x

[b41] Kazmierczak M. J., Wiedmann M. & Boor K. J. Alternative sigma factors and their roles in bacterial virulence. Microbiol Mol Biol Rev. 69, 527–543 (2005). 16339734 10.1128/MMBR.69.4.527-543.2005 PMC1306804

[b42] Song L. *et al.* A genome-wide study of two-component signal transduction systems in eight newly sequenced mutans streptococci strains. BMC genomics. 13, 128 (2012). 22475007 10.1186/1471-2164-13-128 PMC3353171

[b43] van Die I. M., Bergmans H. E. & Hoekstra W. P. Transformation in Escherichia coli: studies on the role of the heat shock in induction of competence. J Gen Microbiol. 129, 663–670 (1983). 6348205 10.1099/00221287-129-3-663

[b44] Murphy M. E. The HSP70 family and cancer. Carcinogenesis. 34, 1181–1188, (2013). 23563090 10.1093/carcin/bgt111 PMC3670260

[b45] Vriezen J. A., de Bruijn F. J. & Nusslein K. Responses of rhizobia to desiccation in relation to osmotic stress, oxygen, and temperature. Appl Environ Microbiol. 73, 3451–3459 (2007). 17400779 10.1128/AEM.02991-06 PMC1932662

[b46] Tao L., Chattoraj P. & Biswas I. CtsR regulation in mcsAB-deficient Gram-positive bacteria. J Bacteriol. 194, 1361–1368 (2012). 22247503 10.1128/JB.06746-11 PMC3294867

[b47] Deng D. M., ten Cate J. M. & Crielaard W. The adaptive response of Streptococcus mutans towards oral care products: involvement of the ClpP serine protease. Eur J Oral Sci. 115, 363–370, (2007). 17850424 10.1111/j.1600-0722.2007.00477.x

[b48] Fleury B. *et al.* Transcriptomic and metabolic responses of Staphylococcus aureus exposed to supra-physiological temperatures. BMC Microbiol. 9, 76, (2009). 19386094 10.1186/1471-2180-9-76 PMC2687450

[b49] Nobbs A. H., Lamont R. J. & Jenkinson H. F. Streptococcus adherence and colonization. Microbiol Mol Biol Rev. 73, 407–450 (2009). 19721085 10.1128/MMBR.00014-09 PMC2738137

[b50] Koo H., Xiao J. & Klein M. I. Extracellular polysaccharides matrix--an often forgotten virulence factor in oral biofilm research. Int J Oral Sci. 1, 229–234 (2009). 20690427 10.4248/IJOS.09086 PMC3733601

[b51] Koo H., Falsetta M. L. & Klein M. I. The exopolysaccharide matrix: a virulence determinant of cariogenic biofilm. J Dent Res. 92, 1065–1073 (2013). 24045647 10.1177/0022034513504218 PMC3834652

[b52] Liu C. *et al.* Hyperosmotic response of streptococcus mutans: from microscopic physiology to transcriptomic profile. BMC Microbiol. 13, 275 (2013). 24289739 10.1186/1471-2180-13-275 PMC4219374

[b53] Hong F. *et al.* RankProd: a bioconductor package for detecting differentially expressed genes in meta-analysis. Bioinformatics. 22, 2825–2827, (2006). 16982708 10.1093/bioinformatics/btl476

[b54] Subramanian A., Kuehn H., Gould J., Tamayo P. & Mesirov J. P. GSEA-P: a desktop application for Gene Set Enrichment Analysis. Bioinformatics. 23, 3251–3253 (2007). 17644558 10.1093/bioinformatics/btm369

[b55] Li M. Y., Huang R. J., Zhou X. D. & Gregory R. L. Role of sortase in Streptococcus mutans under the effect of nicotine. Int J Oral Sci. 5, 206–211 (2013). 24136674 10.1038/ijos.2013.86 PMC3967321

[b56] Taboada B., Verde C. & Merino E. High accuracy operon prediction method based on STRING database scores. Nucleic Acids Res. 38, e130 (2010). 20385580 10.1093/nar/gkq254 PMC2896540

[b57] Xu X., Zhou X. D. & Wu C. D. The tea catechin epigallocatechin gallate suppresses cariogenic virulence factors of Streptococcus mutans. Antimicrob Agents Chemother. 55, 1229–1236 (2011). 21149622 10.1128/AAC.01016-10 PMC3067078

[b58] Cheng L. *et al.* Antibacterial amorphous calcium phosphate nanocomposites with a quaternary ammonium dimethacrylate and silver nanoparticles. Dent Mater. 28, 561–572, (2012). 22305716 10.1016/j.dental.2012.01.005 PMC3322309

[b59] Liao S. *et al.* Streptococcus mutans extracellular DNA is upregulated during growth in biofilms, actively released via membrane vesicles, and influenced by components of the protein secretion machinery. J Bacteriol. 196, 2355–2366 (2014). 24748612 10.1128/JB.01493-14 PMC4054167

[b60] Xu X., Zhou X. D. & Wu C. D. Tea catechin epigallocatechin gallate inhibits Streptococcus mutans biofilm formation by suppressing gtf genes. Arch Oral Biol. 57, 678–683 (2012). 22169220 10.1016/j.archoralbio.2011.10.021

