# Supplementary material for: Streptococcus mutans copes with heat stress by multiple transcriptional regulons modulating virulence and energy metabolism
Source: Sci Rep. 2015 Aug 7;5:12929. doi: 10.1038/srep12929 (PMC4528225; doi:10.1038/srep12929)
Supplement: Supplementary Figures [file srep12929-s1.doc]

*Streptococcus mutans* copes with heat stress by multiple transcriptional regulons modulating virulence and energy metabolism

Chengcheng Liu^1, 4, 5#^, Yulong Niu^3#^, Xuedong Zhou^1, 2^, Xin Zheng^1, 2^, Shida Wang^1^, Qiang Guo^1^, Yuqing Li^1^, Mingyun Li^1^, Jiyao Li^1, 2^, Yi Yang^3^, Yi Ding^1, 4^, Richard J. Lamont^5^, Xin Xu^1, 2*^


^1^State Key Laboratory of Oral Disease, West China Hospital of Stomatology, Sichuan University, Chengdu, PR China
^2^Department of Operative Dentistry and Endodontics, West China Hospital of Stomatology, Sichuan University, Chengdu, PR China
^3^Key Lab of Bio-resources and Eco-environment of Ministry of Education, College of Life Sciences, Sichuan University, Chengdu, PR China
^4^Department of Periodontics, West China Hospital of Stomatology, Sichuan University, Chengdu, PR China
^5^Center for Oral Health and Systemic Disease, School of Dentistry, University of Louisville, Louisville, Kentucky, USA

**
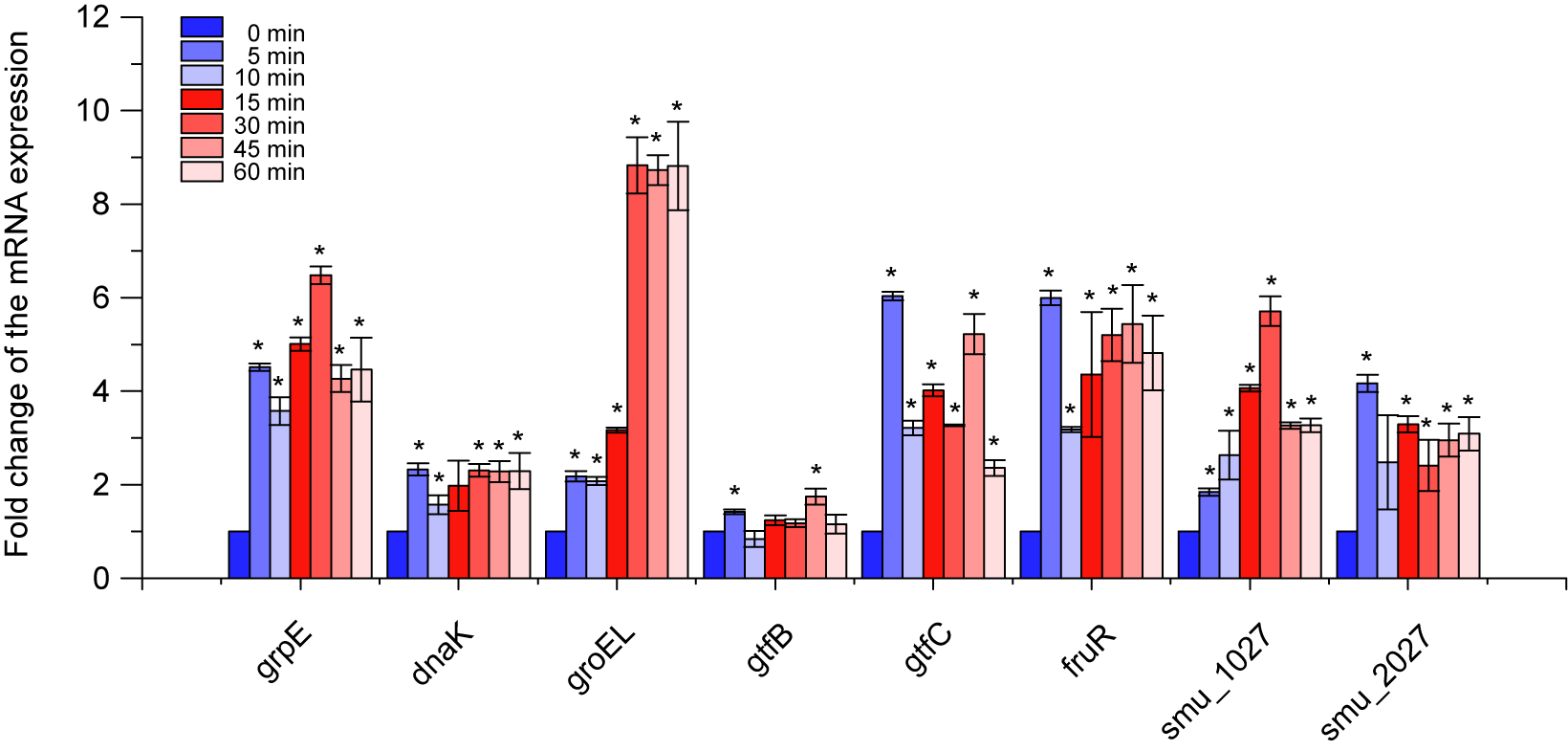
**

**Figure S1 Real-time qPCR validation of the expression of eight selected genes in *S. mutans* ATCC55677^TM^ under heat stress.** Fold change of each gene was relative to control (37℃). Results are presented as mean ± standard deviation (n≥3, *P < 0.05).

**
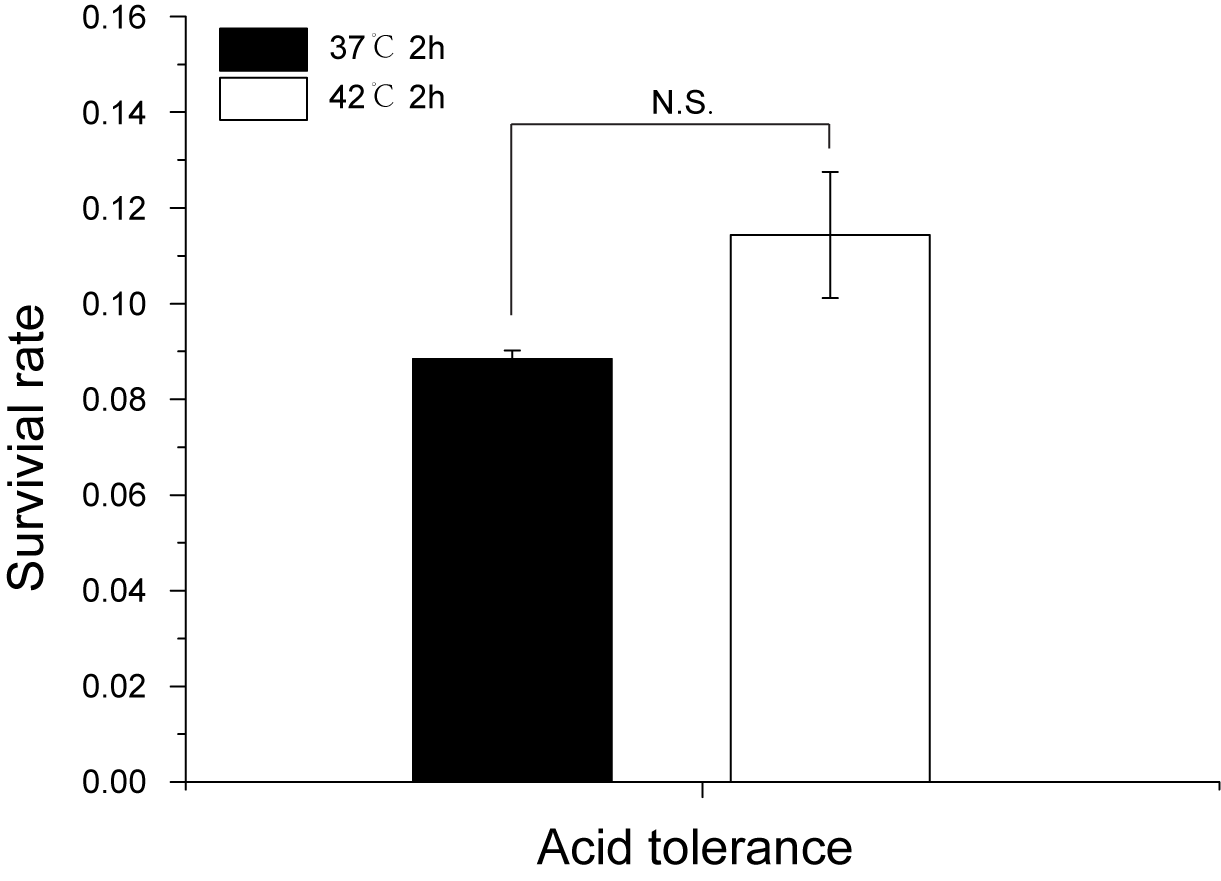
**

**Figure S2 Effect of pre-adaptation to heat stress on the survival of *S. mutans* cells after acid challenge.** Pre-adaptation of *S. mutans* cells to 42℃ had no significant effect on the survival rate of the bacteria challenged by acid. Data are shown as mean ± standard deviation (n≥3). N.S.: no significant difference.
